# Drug Inventory Management in a Pharmacy Store of a Tertiary Care Hospital Using Always Better Control (ABC) and Vital, Essential, and Desirable (VED) Analysis

**DOI:** 10.7759/cureus.110514

**Published:** 2026-06-09

**Authors:** Gopiga Ilango, Anandabaskar Nishanthi, Vivekraj Navabalan, Vimala Ananthy, Shanthi Manickam, Chandrakumar Suryakumar

**Affiliations:** 1 Pharmacology, Sri Manakula Vinayagar Medical College and Hospital, Puducherry, IND; 2 Pharmacology, Mahatma Gandhi Medical College and Research Institute, Puducherry, IND

**Keywords:** abc analysis, abc–ved matrix, drug inventory management, inventory control, pharmacy stores, ved analysis

## Abstract

Background: Efficient drug inventory management ensures the uninterrupted availability of critical medicines while optimizing limited financial resources. Always Better Control (ABC) and Vital, Essential, and Desirable (VED) analyses are validated tools for identifying high-cost and high-priority items requiring strict oversight.

Objective: This study aimed to analyze annual drug procurement expenditure using ABC, VED, and ABC-VED matrix methods in a tertiary care hospital from April 2018 to March 2019 and to identify priority medicines requiring enhanced managerial oversight to optimize inventory control and resource utilization.

Methods: This retrospective study evaluated all drugs purchased over one year. Drugs were categorized using ABC, VED, and ABC-VED matrix analyses (Categories I-III) to identify priority groups for managerial control. Category I comprised AV, AE, AD, BV, and CV items; Category II included BE, BD, and CE items; and Category III consisted solely of CD items.

Results: Overall, 1,402 drugs were procured during the study period, with an annual expenditure of ₹85,305,041.13. ABC analysis classified 135 items (9.62%) as Category A, 250 (17.83%) as Category B, and 1,017 (72.54%) as Category C, accounting for ₹59,813,194.80 (70.11%), ₹17,201,737.31 (20.16%), and ₹8,290,109.02 (9.72%) of total expenditure, respectively. VED analysis identified 373 items (26.60%) as vital, 613 (43.72%) as essential, and 416 (29.67%) as desirable, contributing ₹31,567,217.65 (37.00%), ₹39,230,038.25 (46.00%), and ₹14,507,785.23 (17.00%) of annual costs, respectively. Regarding the ABC-VED matrix, 458 items (32.66%) were classified as Category I, 624 (44.51%) as Category II, and 320 (22.82%) as Category III, accounting for ₹66,958,985.88 (78.49%), ₹15,745,645.07 (18.46%), and ₹2,600,410.18 (3.05%) of total expenditure, respectively.

Conclusion: The ABC-VED matrix is a robust tool for identifying drugs requiring close monitoring and targeted cost-containment strategies while ensuring the continuous availability of vital and essential medicines in resource-limited settings.

## Introduction

Continuous availability of essential medicines is fundamental to safe and effective hospital care, particularly in resource-constrained tertiary hospitals where drug budgets are limited, and demand is high. Effective treatment depends on an adequate and continuous supply of drugs. Prior research shows that medicine procurement alone accounts for 30%-35% of total hospital expenditure [1]. Therefore, efficient inventory management is essential to ensure the optimal utilization of limited healthcare resources.

Several inventory management techniques have been developed to optimize pharmaceutical procurement and stock control. Commonly used methods include Always Better Control (ABC) analysis, which classifies items according to expenditure; Vital, Essential, and Desirable (VED) analysis, which prioritizes medicines based on clinical criticality; Fast-moving, Slow-moving, Non-moving (FSN) analysis, which evaluates consumption patterns; and XYZ analysis, which categorizes items according to inventory value. Matrix approaches such as ABC-VED combine cost and criticality considerations to identify medicines requiring different levels of managerial control and monitoring [2].

Recent advances in digital technologies have further strengthened hospital inventory management. Computerized inventory systems, automated dispensing technologies, and electronic pharmacy management platforms enable real-time stock monitoring, improve ordering accuracy, reduce stock-outs and overstocking, minimize wastage due to expired medicines, and support data-driven procurement decisions. Integration of such digital systems with established inventory control tools, such as ABC-VED analysis, can enhance inventory visibility and operational efficiency in hospital pharmacies [3]. Thus, pharmacy stores must be managed judiciously to ensure efficient clinical and administrative services.

ABC analysis categorizes drugs based on annual consumption value into Categories A, B, and C. Category A comprises 10% of drugs but accounts for nearly 70% of total expenditure, requiring strict day-to-day monitoring. Category B includes approximately 20% of drugs, contributing nearly 20% of total expenditure and requiring periodic review. Category C includes 70% of drugs, accounting for 10% of total expenditure and requiring minimal review [4]. However, ABC analysis is limited because it considers only the monetary value and quantity of drug consumption without accounting for drug criticality. This limitation can be addressed using VED analysis, which classifies drugs as Vital, Essential, or Desirable [4]. Vital drugs are lifesaving and indispensable for treatment; Essential drugs can be omitted briefly without major impact; and desirable drugs can be omitted for longer periods. Combining ABC and VED analyses (ABC-VED matrix) enables the identification of drugs with the greatest economic impact and prioritizes critical medicines for effective patient care [5].

Several studies have applied ABC and VED analyses to drug inventory management, enabling the prioritization of drugs requiring strict control in hospital settings [6-10].

Our tertiary care teaching hospital has over 900 inpatient beds and serves patients across multiple medical and surgical specialties. The hospital pharmacy procures 1,000-1,200 drugs annually for both inpatient and outpatient use. Effective inventory management techniques are therefore essential to ensure a consistent drug supply and prevent stock-outs. Thus, this study aimed to analyze annual drug procurement expenditure using ABC, VED, and ABC-VED matrix methods in a tertiary care hospital from April 2018 to March 2019 and to identify priority medicines requiring enhanced managerial oversight to optimize inventory control and resource utilization.

## Materials and methods

This retrospective cross-sectional study was conducted over two months (July-August 2019) at a tertiary care hospital in the Departments of Pharmacy and Pharmacology. Institutional Human Ethics Committee approval was obtained before study initiation (EC/24/2019). A consent waiver was granted by the Ethics Committee. Data confidentiality was maintained, and the study adhered to the principles of the Declaration of Helsinki. Prior permission was obtained from the Department of Pharmacy. Data on drugs procured (excluding vaccines, IV fluids, and blood products) during April 2018-March 2019, including annual demand and unit cost, were collected from the pharmacy database using a structured form. Data were entered into a Microsoft Excel (Microsoft Corporation, Redmond, WA, USA) spreadsheet and analyzed using descriptive statistics. Categorical variables are presented as frequencies and percentages, and drug procurement costs are reported in Indian Rupees.

Always Better Control analysis

Annual drug expenditure was calculated by multiplying annual drug demand by unit cost. Expenditures were then ranked in descending order, and cumulative costs and percentages of expenditure and items were calculated. Based on annual expenditure, drugs were classified into Categories A, B, and C. Drugs accounting for the top 70% of total expenditure were assigned to Category A, the next 20% to Category B, and the remaining 10% to Category C [4].

All drug procurement records available in the pharmacy database during the study period, including emergency purchases and drug stock-outs, were included in the analysis. Any supplier-related drug price variations were inherently reflected in the procurement expenditure data used for ABC analysis.

Vital, Essential, and Desirable analysis

Drugs were classified into three categories: vital (V), essential (E), and desirable (D). Lifesaving drugs requiring uninterrupted availability were classified as vital; those that could be temporarily omitted were classified as essential; and the remaining drugs were classified as desirable. This classification was performed by an institutional pharmacy committee comprising the Medical Superintendent, Chief Pharmacist, Nursing Superintendent, and Heads of the Departments of Pharmacology, General Medicine, General Surgery, Obstetrics and Gynecology, Pediatrics, and Emergency Medicine. Decisions were guided by the hospital formulary, specialty-specific consensus, and the frequency of use in emergency settings, with final categorization determined through majority voting. Annual drug expenditure and its percentage distribution across categories were also calculated [4].

Always Better Control-Vital, Essential, and Desirable matrix analysis

An ABC-VED matrix was constructed by combining ABC and VED results. All drugs were classified into nine subgroups: AV, BV, CV, AE, BE, CE, AD, BD, and CD, where the first and second letters represent the ABC and VED categories, respectively. These were further consolidated into three categories: Category I (AV, BV, CV, AE, and AD), Category II (BE, CE, and BD), and Category III (CD). Annual drug expenditure and its percentage distribution across categories were also calculated [6,7,11].

## Results

Overall, 1,402 drugs were procured over one year (April 2018-March 2019). The total annual expenditure for these drugs was ₹85,305,041.13. 

Always Better Control analysis

**Figure 1:**
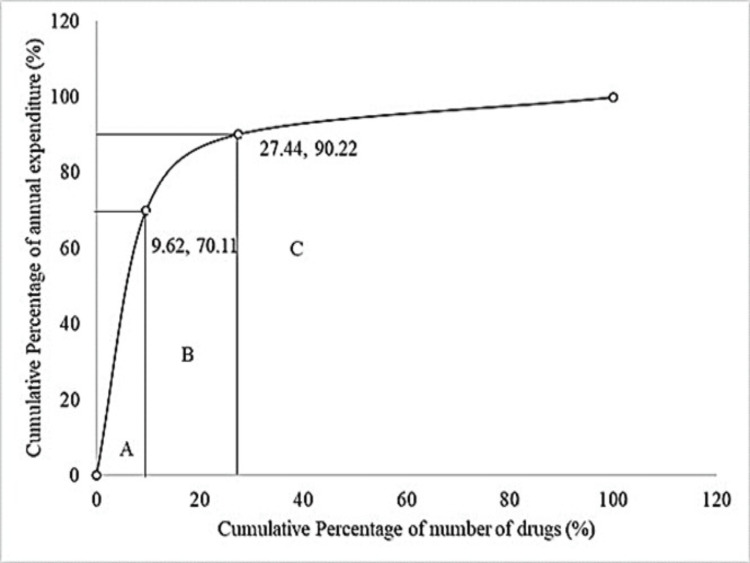
Cumulative curve of ABC analysis The figure depicts the cumulative percentage of annual drug expenditure plotted against the cumulative percentage of drug items arranged in descending order of expenditure. ABC, Always Better Control

ABC analysis classified 135 (9.62%) items as Category A, 250 (17.83%) as Category B, and 1,017 (72.54%) as Category C. These categories accounted for ₹59,813,194.80 (70.11%), ₹17,201,737.31 (20.16%), and ₹8,290,109.02 (9.72%) of total expenditure, respectively (Table 1; Figure 1).

**Table 1:** ABC, VED, and ABC-VED matrix analysis of the pharmacy store in a tertiary care hospital, 2018-2019 ABC, Always Better Control; VED, Vital, Essential, and Desirable

Type of analysis	Category	No of items (N=1402)	Percentage of items (%)	Annual drug expenditure	Percentage of annual drug expenditure of the pharmacy (%)
ABC analysis	A	135	9.62%	59,813,194.80	70.11%
	B	250	17.83%	17,201,737.31	20.16%
	C	1,017	72.54%	8,290,109.02	9.72%
VED analysis	V	373	26.60%	31,567,217.65	37%
	E	613	43.72%	39,230,038.25	46%
	D	416	29.67%	14,507,785.23	17%
ABC-VED matrix analysis	I	458	32.66%	66,958,985.88	78.49%
	II	624	44.51%	15,745,645.07	18.46%
	III	320	22.82%	2,600,410.18	3.05%

Vital, Essential, and Desirable analysis

**Figure 2:**
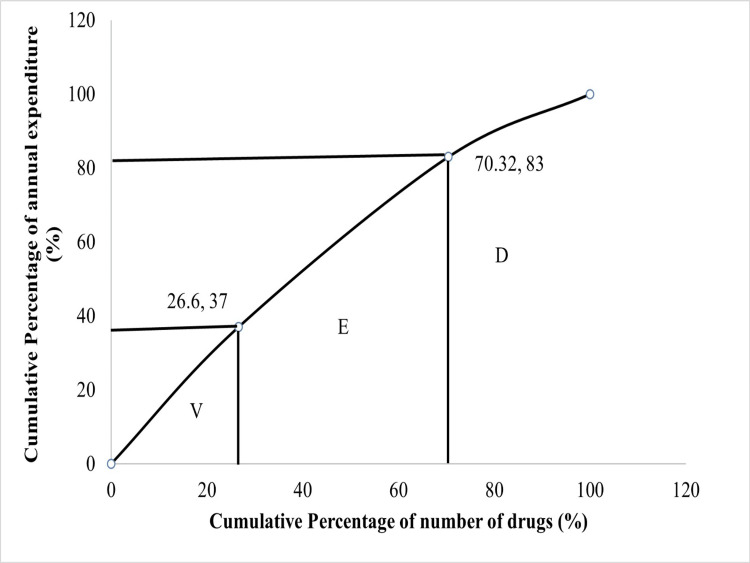
Cumulative curve of VED analysis The figure depicts the cumulative percentage of annual drug expenditure plotted against the cumulative percentage of drug items classified according to their clinical criticality. VED, Vital, Essential, and Desirable

VED analysis classified 373 (26.6%) items as vital, 613 (43.72%) as essential, and 416 (29.67%) as desirable. These categories contributed ₹31,567,217.65 (37%), ₹39,230,038.25 (46%), and ₹14,507,785.23 (17%) of the total annual expenditure, respectively (Table 1; Figure 2).

Always Better Control-Vital, Essential, and Desirable matrix analysis

**Figure 3:**
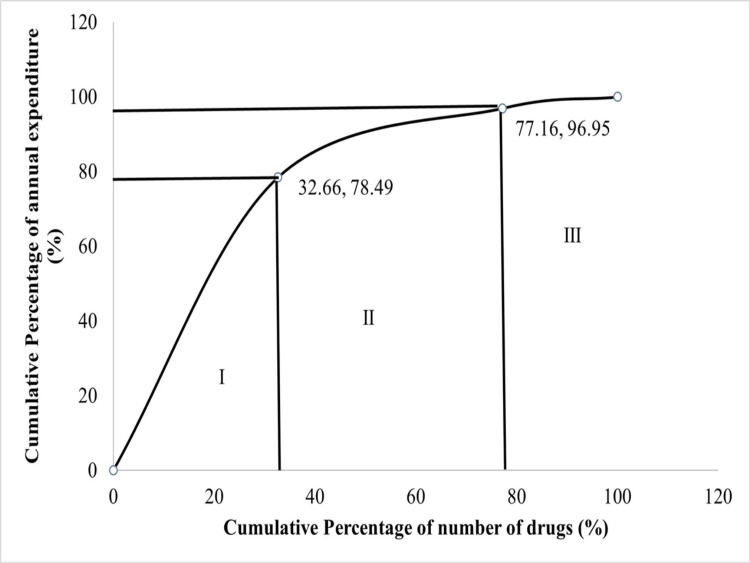
Cumulative curve of the ABC-VED matrix analysis The figure illustrates the cumulative distribution of annual drug expenditure against the cumulative percentage of drug items across Categories I, II, and III of the ABC-VED matrix. ABC, Always Better Control; VED, Vital, Essential, and Desirable

The ABC-VED matrix classified 458 (32.66%) items into Category I, 624 (44.51%) into Category II, and 320 (22.82%) into Category III (Figure 3). These categories accounted for ₹66,958,985.88 (78.49%), ₹15,745,645.07 (18.46%), and ₹2,600,410.18 (3.05%) of total expenditure, respectively (Table 1; Figure 3). In Category I, the numbers of items in AV, BV, CV, AE, and AD were 50 (3.57%), 71 (5.06%), 252 (17.97%), 65 (4.64%), and 20 (1.43%), respectively. Within Category II, BE, CE, and BD accounted for 103 (7.35%), 445 (31.74%), and 76 (5.42%) items, respectively, while Category III (CD) accounted for 320 items (22.82%) (Table 2).

**Table 2:** ABC-VED matrix analysis of the pharmacy store in a tertiary care hospital, 2018-2019 ABC, Always Better Control; VED, Vital, Essential, and Desirable

Drug category	V			E			D		
	Combined category	No. of items	Percentage of items (%)	Combined category	No. of items	Percentage of items (%)	Combined category	No. of items	Percentage of items (%)
A	AV	50	3.57	AE	65	4.64	AD	20	1.43
B	BV	71	5.06	BE	103	7.35	BD	76	5.42
C	CV	252	17.97	CE	445	31.74	CD	320	22.82

## Discussion

This study showed that one-third of items accounted for nearly four-fifths of annual expenditure, confirming a marked concentration of spending. The ABC-VED matrix further identified a manageable subset of high-priority items requiring intensified control. Table 3 compares our study findings with those of previously published studies, providing context within the existing literature.

**Table 3:** Comparison of ABC-VED matrix analysis of various studies ABC, Always Better Control; VED, Vital, Essential, and Desirable; EPSA, Ethiopian pharmaceutical supply agency

Author name (year of publication)	Study setting (country)		Percentage of items (%)								
			ABC analysis			VED analysis			Categories in ABC-VED analysis		
			A	B	C	V	E	D	I	II	III
Present study	Tertiary care hospital (900-bedded) (India)		9.62	17.83	72.54	26.60	43.72	29.67	32.66	44.51	22.82
Jobira T, et al. (2021) [2]	One tertiary care hospital and 13 health centers (Ethiopia)		12.1	10.2	77.7	16.9	67.9	15.2	26.6	49.2	24.2
Kumar S, et al. (2014)[6]	Tertiary care hospital (India)		6.77	19.27	73.95	13.14	56.37	30.49	21	51.17	27.83
Devnani M, et al. (2010) [7]	Tertiary care hospital (India)		13.78	21.85	64.37	12.11	59.38	28.51	22.09	54.63	23.28
Meena DK, et al. (2025)[10]	One tertiary care hospital and nine primary health care centers (India)	State govt.	7.8	19.3	72.8	26.4	47.8	25.7	32.1	50.0	17.8
		Central govt.	10.4	14.9	74.7	27.3	55.8	16.9	37.0	53.2	9.7
		Tertiary care hospital	15.4	22.2	62.4	25.8	40.6	33.5	36.1	44.2	19.6
Gizaw T, et al. (2021) [11]	EPSA Jimma hub, governmental organization (393 pharmaceuticals in Ethiopia)		14.8	19.8	65.4	42	49	9	47.58	45.29	7.13
Taser M, et al. (2024)[12]	University Hospital (Turkey)		5.43	11.15	83.42	37.23	43.33	19.44	38.66	42.65	18.69
Verma H, et al. (2025) [13]	Tertiary care hospital (India)		12.89	23.12	64	23.56	52.89	23.56	41.78	44.89	13.34
Jaju R, et al. (2023) [14]	Tertiary care hospital (newly started) (India)		22.80	24.06	53.14	6.82	60.56	32.62	26.0	55.85	17.99
Dhodi D, et al. (2021) [15]	Tertiary care teaching hospital (India)		14.37	21.40	64.21	24.28	52.71	23.00	36.10	49.20	14.69
Mohammed SA, et al. (2020) [16]	Tertiary care hospital (Ethiopia)		17	20.17	62.83	34.56	63.74	1.7	43.68	54.79	1.53

ABC analysis classified medicines according to their contribution to annual drug expenditure. In this study, Category A comprises 135 items (9.62%) of the total inventory but accounted for 70.11% of annual drug spending, consistent with the classical Pareto principle and highlighting the need for stringent monitoring of this small but high-value subset. Categories B and C represented 250 (17.83%) and 1,017 items (72.54%), contributing 20.16% and 9.72% of total expenditure, respectively. Previous studies report that Category A drugs typically range from 5.43% to 22.80% of total items [2,6,7,10-16]. This variation reflects differences in procurement patterns and utilization of high-cost medicines; centers with a greater concentration of expensive drugs may exhibit a lower proportion of Category A items.

In our study, VED analysis, which prioritizes criticality rather than cost, revealed that the vital (V) category comprised 373 items (26.6%) of the inventory and accounted for 37% of total expenditure, while the essential (E) category comprised 613 items (43.72%) and accounted for 46% of expenditure. The desirable (D) category comprised 416 items (29.67%) and accounted for only 17% of total expenditure. Previous studies report that V category drugs range from 6.82% to 42% of total items [2,6,7,10-16]. This variability in V-category proportions across studies may reflect differences in the scope of specialized clinical services offered by institutions and variations in the underlying disease burden of the populations they serve.

The ABC-VED matrix analysis, which integrates cost and criticality, further refines the prioritization of medicines. Category I, comprising all vital items and high-value drugs (AV, AE, AD, BV, and CV), constituted 458 items (32.66%) of the inventory but accounted for 78.49% of total expenditure, a proportion consistent with that of previous reports in which Category I drugs ranged from 21% to 47.58% [2,6,7,10-16]. These findings indicate that prioritizing the 458 Category I items out of 1,402 drugs is essential to maximize pharmacy operational efficiency. Within Category I, AV, BV, and CV formed a core set of drugs indispensable for uninterrupted patient care. Despite varying cost profiles, these vital medicines require stringent oversight because stock-outs can directly compromise clinical outcomes, particularly in emergency, critical-care, and high-dependency settings. The AE and AD subgroups both include high-expenditure medicines; however, AD items are therapeutically desirable rather than essential and constituted 1.43% of the total inventory. Given their non-critical clinical role despite substantial cost implications, AD medicines represent an appropriate target for cost-containment strategies, including rational prescribing audits, optimization of procurement prices, and evaluation of suitable therapeutic alternatives, without adversely affecting patient care.

Category II (BE, BD, and CE), accounting for 624 items (44.51%) of the inventory and 18.46% of annual expenditure, requires moderate managerial oversight. BE drugs, which are clinically essential and of moderate cost, require regular monitoring to ensure uninterrupted availability, while CE drugs, although low-cost, should be controlled to prevent unnecessary accumulation and wastage. Within this category, BD drugs, contributing 6.22% of total expenditure, warrant periodic review to minimize overstocking, expiry, and procurement inefficiencies associated with fluctuating demand. Previous studies report Category II proportions ranging from 42.65% to 55.85%, indicating consistency with the existing literature [2,6,7,10-16].

Category III, consisting exclusively of CD drugs, comprised 320 items (22.82%) of the inventory and accounted for 3.05% of annual expenditure. Although these medicines contribute minimally to overall expenditure, their large number increases the risk of overstocking, expiry, and inefficient use of storage space, underscoring the need for prudent inventory control and avoidance of unnecessary procurement. Previous studies report that the proportion of CD drugs ranges from 1.53% to 27.83%, thereby placing the present findings within the range documented in earlier studies [2,6,7,10-16].

In our study, the ABC-VED matrix helped identify drugs requiring stringent control and those that can be managed with less intensive supervision. Category I drugs, which accounted for the majority of annual drug expenditure, require enhanced managerial oversight because of their high cost and/or clinical importance. The AD group, comprising high-cost but therapeutically desirable medicines, emerged as a major contributor to avoidable expenditure and therefore warrants close monitoring. Vital drugs, irrespective of cost, should remain continuously available to prevent interruptions in patient care. Although CD drugs account for only a small proportion of total expenditure, avoiding their unnecessary overstocking may contribute to more efficient utilization of limited healthcare resources. Overall, the ABC-VED matrix provides a practical framework for inventory prioritization and may support rational procurement planning, effective inventory control, and optimal allocation of healthcare resources in hospital pharmacies.

Limitations

This study has some limitations. First, it was conducted in a single tertiary care hospital; therefore, the findings may not fully represent drug-use patterns in other healthcare settings. Second, changes in drug prescribing practices and their influence on drug procurement patterns could not be evaluated separately. Furthermore, although VED classification was performed by a multidisciplinary committee using consensus-based decision-making, some degree of subjectivity is inherent in the categorization process.

## Conclusions

Our study shows that ABC-VED matrix analysis is a practical tool for identifying a small group of high-priority medicines that require close monitoring. Focused monitoring of Category I drugs may support better inventory control, rational procurement planning, and optimal utilization of available resources in tertiary care hospitals. Future prospective studies may provide more comprehensive insights into inventory management practices. In addition, the integration of ABC-VED analysis with computerized inventory monitoring systems that enable real-time stock tracking, automated procurement alerts, and data-driven inventory control may further strengthen inventory management.
